# Peptides from *Harpadon nehereus* Bone Ameliorate Sodium Palmitate-Induced HepG2 Lipotoxicity by Regulating Oxidative Stress and Lipid Metabolism

**DOI:** 10.3390/md23030118

**Published:** 2025-03-09

**Authors:** Siyi Song, Wei Zhao, Qianxia Lin, Jinfeng Pei, Huoxi Jin

**Affiliations:** School of Food and Pharmacy, Zhejiang Ocean University, Zhoushan 316022, China; song18234214689@163.com (S.S.); zhaoweiolivia@163.com (W.Z.); linqianxia_zjou@163.com (Q.L.)

**Keywords:** sodium palmitate, HepG2, hyperlipidemia, oxidative stress, lipid metabolism

## Abstract

Antioxidant peptides are a well-known functional food exhibiting multiple biological activities in health and disease. This study investigated the effects of three peptides, LR-7 (LALFVPR), KA-8 (KLHDEEVA), and PG-7 (PSRILYG), from *Harpadon nehereus* bone on sodium palmitate (PANa)-induced HepG2. The findings indicated that all three peptides significantly reduced the oxidative damage and fat accumulation in the HepG2 cells while also normalizing the abnormal blood lipid levels caused by PANa. Furthermore, treatment with LR-7 resulted in a more than 100% increase in catalase (CAT), glutathione peroxidase (GSH-Px), and nuclear factor erythroid 2-related factor 2 (Nrf2) levels within the HepG2 cells (*p* < 0.001). Western blot analysis showed that LR-7 treatment significantly lowered the expression of fatty acid synthase (FASN) by 59.6% (*p* < 0.001) while enhancing carnitine palmitoyl transferase 1 (CPT1) by 134.7% (*p* < 0.001) and adipose triglyceride lipase (ATGL) by 148.1% (*p* < 0.001). Additionally, these peptides effectively inhibited the pancreatic lipase activity. Notably, LR-7 demonstrated superior effectiveness across all of the evaluated parameters, likely due to its greater hydrophobicity. In summary, LR-7, KA-8, and PG-7 are effective at mitigating oxidative stress as well as regulating lipid metabolism, thus protecting HepG2 cells from PANa-induced injury and lipid buildup. This research indicates that these collagen-derived peptides, especially LR-7, show promise as natural agents for managing hyperlipidemia.

## 1. Introduction

Lipotoxicity is a pathological feature of chronic liver disease. It is characterized by the accumulation of lipids, which in turn leads to cell dysfunction and injury [1]. Saturated fatty acids (SFAs), as essential dietary components, play a crucial role in maintaining human physiological structure and function. However, excessive circulating lipids and subsequent cellular uptake can induce lipotoxicity, which has been implicated in the pathogenesis of various metabolic disorders and contributes to the progression of non-alcoholic fatty liver disease (NAFLD) [2,3]. Distinct effects on cell survival and death are exerted by saturated versus unsaturated fatty acids; however, the mechanisms behind these differences remain poorly understood. Numerous studies have shown that free fatty acids can lead to liver cell damage and apoptosis [4]. High concentrations of free fatty acids result in fat accumulation within the liver, prompting inflammatory factor release and cellular dysfunction [5]. Palmitic acid (PA), a prevalent saturated fatty acid constituting about 20–30% of total body fats, can be ingested through diet or synthesized endogenously via de novo lipogenesis (DNL) [6]. PA ranks among the most abundant saturated fatty acids found in plasma and has been implicated in toxicity affecting pancreatic beta cells as well as hepatocytes, among other cell types [7]. HepG2 cells are frequently utilized in vitro to study hyperlipidemia due to their capacity to accumulate intracellular lipids when exposed to free fatty acids like oleic acid and palmitic acid [8].

Numerous studies have shown that oxidative stress is a primary mechanism responsible for inducing abnormal lipid metabolism [9]. Oxidative stress causes lipid peroxidation and the formation of oxidized low-density lipoprotein (ox-LDL), which exacerbates hyperlipidemia [10]. Elevated blood lipid levels, combined with the buildup of lipid metabolism by-products, contribute to the onset of oxidative stress [11]. A promising approach to managing hyperlipidemia involves reducing the blood lipid and oxidative stress levels. Recent studies have highlighted the potential role of food protein peptides in treating hyperlipidemia [12]. Evidence suggests that peptides derived from animal proteins exert anti-lipidemic and antioxidant activity [13]. These beneficial effects are partly attributed to the specific amino acid compositions of the peptides [14]. For instance, peptides rich in hydrophobic amino acids, such as leucine, isoleucine, and proline, have shown efficacy in combating oxidative stress and hyperlipidemia [15]. Due to its high proline content, collagen is likely to exhibit significant antioxidant and anti-hyperlipidemic activity. Marine collagen peptides have gained significant attention as a research focus because of their diverse bioactive functions, safety, and lack of toxicity [13,16]. Collagen peptides derived from the skin of the great hammerhead shark (*Sphyrna mokarran*) have been found to mitigate hyperlipidemia by enhancing antioxidant enzyme activities and downregulating the expression of fatty acid synthase and 3-hydroxy-3-methylglutaryl-CoA reductase [17]. However, there is still limited research on the hypolipidemic activities of peptides derived from marine bone collagen and their underlying mechanisms.

*Harpadon nehereus*, a nutrient-rich marine species indigenous to the Indo-Pacific region, remains underexploited despite its significant collagen content (18–22% wet weight) [18]. *Harpadon nehereus* serves as an economical source that is rich in protein and trace elements. Its proteins and derived peptides offer numerous health benefits including immune enhancement, blood pressure reduction, cardiovascular protection, and inflammation mitigation [19]. In prior research, we successfully isolated three novel peptides LALFVPR (LR-7), KLHDEEVA (KA-8), and PSRILYG (PG-7) from the enzymatic hydrolysates of *Harpadon nehereus* bone collagen [20]. Notably, PG-7 was observed to improve angiotensin II-induced dysfunctions within HUVECs [21], while LR-7 exhibited protective qualities against cardiovascular injuries in hypertensive mice [22]. However, the anti-hyperlipidemic activity of these three peptides remain unreported. This study aimed to investigate the antioxidant and lipid metabolism regulatory effects of these peptides on HepG2 cells induced by sodium palmitate (PANa) to assess their potential utility in combating hyperlipidemia.

## 2. Results

### 2.1. Cytoprotective Effects of LR-7, KA-8, and PG-7 on HepG2 Cells

**Figure 1:**
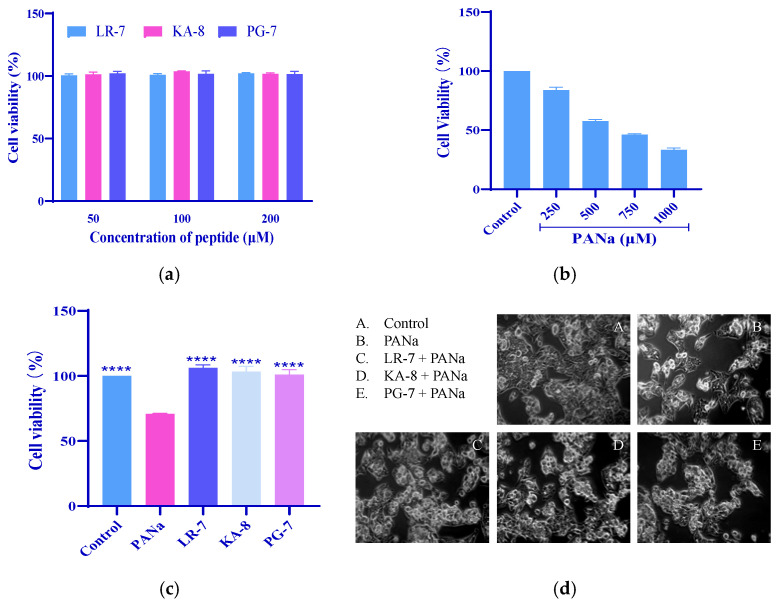
Protective effects of LR-7, KA-8, and PG-7 on HepG2 cells. (**a**) HepG2 cell proliferation after three oligopeptide (50, 100, 200 μM) treatments. (**b**) Effects of PANa on HepG2 cell viability. (**c**) Effects of three peptides (100 µM) on the cell viability of HepG2 induced by 350 µM PANa. (**d**) Morphology of HepG2 (200×). Con: normal control; PANa: HepG2 was treated with 350 µM PANa for 24 h. HepG2 was incubated with 100 µM KA-8 (KA-8 group), LR-7 (LR-7 group), and PG-7 (PG-7 group) for 4 h, followed by 350 µM PANa treatment for 24 h. **** represents *p* < 0.0001 compared with the PANa group.

The impact of peptides LR-7, KA-8, and PG-7 on the viability of HepG2 cells stimulated by PANa was examined. To investigate whether peptides KA-8, LR-7, and PG-7 affected cell apoptosis, HepG2 cells were treated with different concentrations of each peptide (50, 100, 200 µM) for 24 h. As shown in Figure 1a, compared with those of the untreated cells with peptides, the cell proliferation rates were not significantly reduced with the three peptide treatments at concentrations up to 200 µM for 24 h. These results indicated that peptides KA-8, LR-7, and PG-7 had no effect on the apoptosis of HepG2 cells at concentrations below 200 µM. Based on systematic evaluation, a concentration of 100 μM was selected for all three oligopeptides (KA-8, LR-7, and PG-7) in subsequent experimental procedures. As illustrated in Figure 1b, the cell viability decreased from 100% at 0 μM PANa to 33.3% (*p* < 0.0001) at 1000 μM. Based on a comprehensive evaluation, a concentration of 350 μM, an intermediate between 250 and 500 μM, was selected as the optimal concentration for model establishment. Figure 1c demonstrates that treatment with LR-7, KA-8, and PG-7 notably enhanced the cell viability in HepG2 cells exposed to 350 µM PANa. To directly evaluate the protective effects of these peptides on the PANa-treated HepG2 cells, their morphology was observed under a microscope. In Figure 1d, it can be seen that the normal cells (Con) adhered well to the surface and displayed a polygonal shape. Conversely, those in the PANa group appeared loose, with a marked decrease in cell count and showed signs of apoptosis. However, following treatment with all three peptides, clusters formed among the cells along with a considerable increase in their numbers.

### 2.2. Effects of LR-7, KA-8, and PG-7 on Antioxidant Capacity of HepG2 Cells

**Figure 2:**
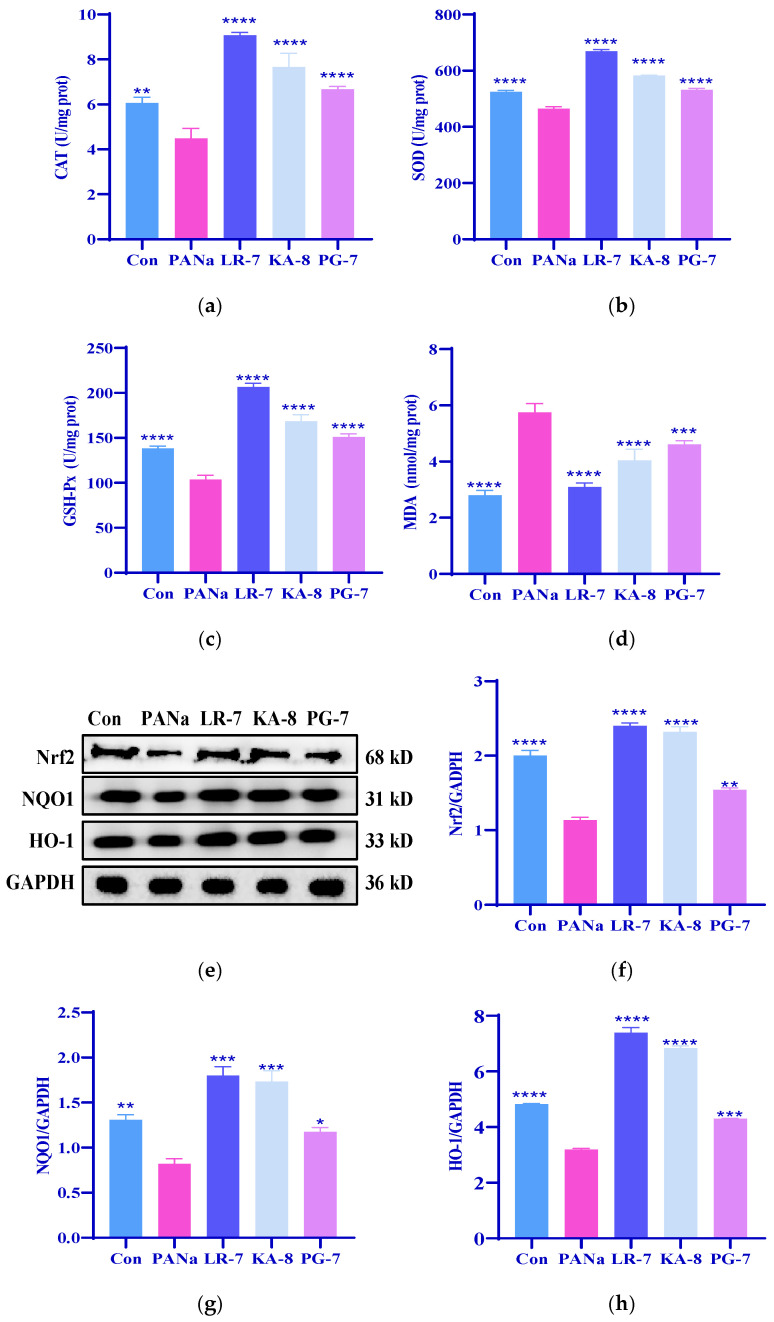
Effects of LR-7, KA-8, and PG-7 on the antioxidant capacity of HepG2 cells. The levels of CAT (**a**), SOD (**b**), GSH-Px (**c**), MDA (**d**), Nrf2 (**e**,**f**), NQO1 (**e**,**g**), and HO-1 (**e**,**h**) in HepG2 cells were measured. Con: normal control; PANa: HepG2 was treated with 350 µM PANa for 24 h. HepG2 was incubated with 100 µM KA-8 (KA-8 group), LR-7 (LR-7 group), and PG-7 (PG-7 group) for 4 h, followed by 350 µM PANa treatment for 24 h. *, **, ***, and **** represent *p* < 0.05, <0.01, <0.001, and <0.0001 compared with the PANa group, respectively.

The mitigation of PANa-induced HepG2 damage by the three peptides in Figure 1 may be related to their reduction of oxidative stress. Research indicates that intracellular antioxidant enzymes play vital roles protecting against oxidative stress damage [23]. To evaluate the antioxidant potential of LR-7, KA-8, and PG-7, we examined their impact on the levels of catalase (CAT), glutathione peroxidase (GSH-Px), superoxide dismutase (SOD), and malondialdehyde (MDA) in the HepG2 cells. Figure 2 illustrates that the activities of CAT and GSH-Px were significantly diminished by more than 20% (*p* < 0.001) in the PANa group when compared with the Con group, while the MDA levels showed a notable increase by 105% (*p* < 0.0001). Treatment with LR-7, KA-8, and PG-7 effectively reversed the reduction in CAT, SOD, and GSH-Px activities induced by PANa. Remarkably, in the LR-7 (100 μM) treatment group, the CAT and GSH-Px activities increased by over 100% than those in the PANa group (*p* < 0.0001). Furthermore, the MDA concentrations within this same group decreased to values similar to those observed in the Con group. These findings indicate that all three peptides, especially LR-7, substantially improved the antioxidant enzyme activity in the HepG2 cells affected by PANa exposure.

Nuclear factor erythroid 2-related factor 2 (Nrf2) is a critical transcription factor that regulates the expression of antioxidant enzymes and plays an essential role in disease prevention. To assess whether LR-7, KA-8, and PG-7 could activate the Nrf2 signaling pathways within the HepG2 cells, we analyzed the protein expressions related specifically to Nrf-2, HO-1, and NQO-1. Figure 2e–h revealed a significant reduction regarding the expression levels of these proteins in the PANa group compared with the Con group. In contrast, following treatment with LR-7, the levels of Nrf2, HO-1, and NQO1 were significantly elevated by 111.5%, 131.7%, and 120% (*p* < 0.0001), respectively. These findings suggest that LR-7, KA-8, and PG-7 enhanced the antioxidant activity of PANa-induced HepG2 by activating the Nrf2 signaling pathway.

### 2.3. Effects of LR-7, KA-8, and PG-7 on Levels of Lipid in HepG2

**Figure 3:**
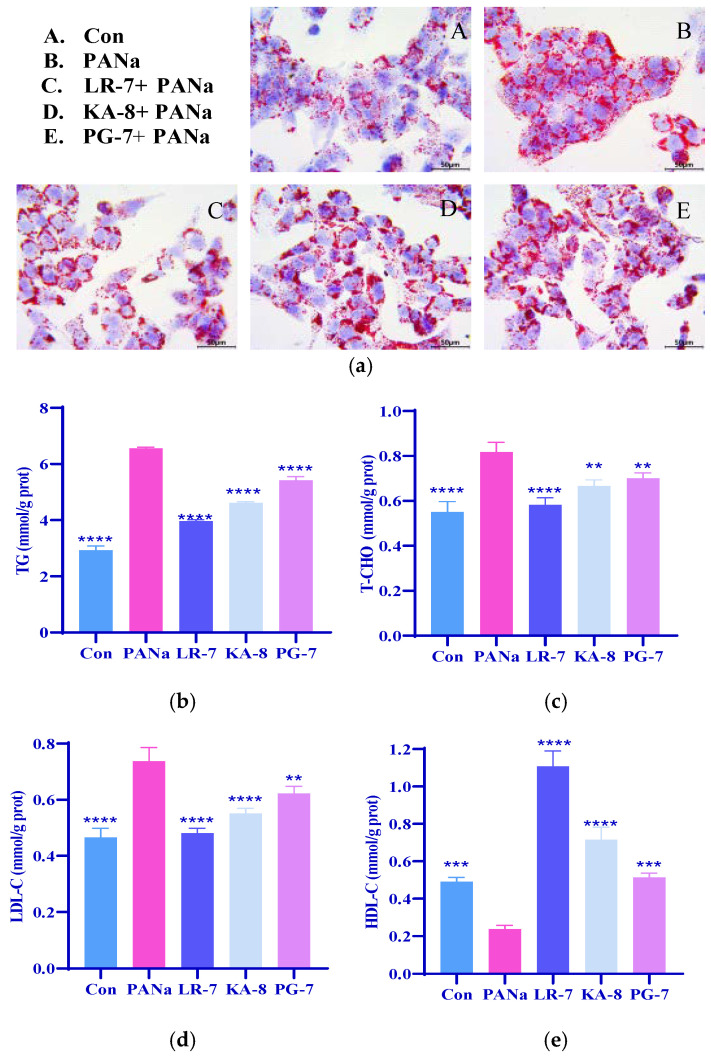
Effects of LR-7, KA-8, and PG-7 on the fat accumulation ((**a**), 200×) and levels of TG (**b**), TCHO (**c**), LDL-C (**d**), and HDL-C (**e**) in the HepG2 cells. Con: normal control; PANa: HepG2 was treated with 350 µM PANa for 24 h. HepG2 was incubated with 100 µM KA-8 (KA-8 group), LR-7 (LR-7 group), and PG-7 (PG-7 group) for 4 h, followed by 350 µM PANa treatment for 24 h. **, ***, and **** represent *p* < 0.01, <0.001, and <0.0001 compared with the PANa group, respectively.

In addition to reducing oxidative stress, lowering blood lipids is at the core of treating hyperlipidemia. Therefore, we investigated the effects of the three peptides on the fat accumulation and lipid levels in PANa-treated HepG2. Oil Red O (ORO) is a fat-soluble dye specifically used for staining neutral fats like triglycerides, resulting in red-stained lipid droplets within the stained cells [24]. As shown in Figure 3a, compared with the Con group, there was an evident rise in fat droplet formation and accumulation within the PANa group. Among the treatments with these three peptides, LR-7 exhibited the most significant reduction in fat accumulation relative to the PANa group. These findings suggest that all three peptides, especially LR-7, effectively diminished the intracellular fat droplet buildup within the HepG2 cells induced by PANa.

Triacylglycerol (TG), total cholesterol (TCHO), low-density lipoprotein cholesterol (LDL-C), and high-density lipoprotein cholesterol (HDL-C) are standard indicators for lipids. To analyze the negative effects of LR-7, KA-8, and PG-7 on the lipid levels in the PANA-induced HepG2, the TG, TCHO, LDL-C, and HDL-C levels were measured, respectively. Figure 3b–e revealed that the TG, TCHO, and LDL-C levels in the PANa group were significantly increased by 124.1%, 48.4%, and 58.3% (*p* < 0.0001), respectively, while HDL-C decreased markedly by 51.7% (*p* < 0.001) when compared with the Con group. Treatment with LR-7, KA-8, and PG-7 notably reversed the PANa-induced increases in TG, TCHO, and LDL-C, and the decrease in HDL-C. Among the three peptides, LR-7 demonstrated the most effective lipid-lowering impact.

### 2.4. Effects of LR-7, KA-8, and PG-7 on the Lipid Metabolism in PANa-Induced HepG2 Cells

**Figure 4:**
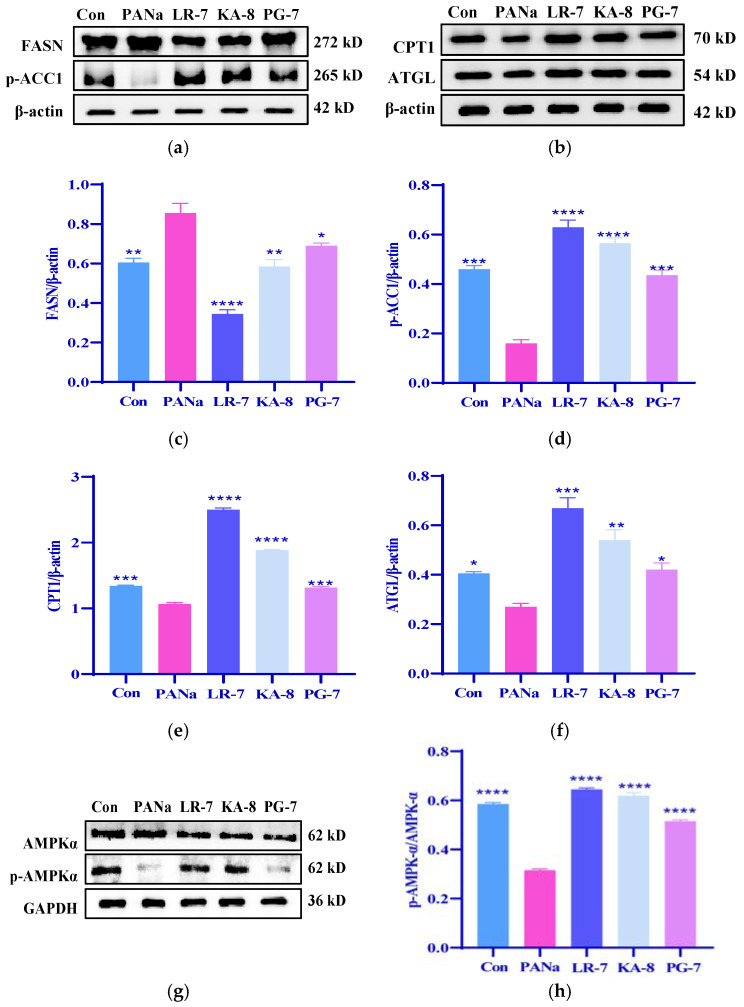
Effects of LR-7, KA-8, and PG-7 on the lipid metabolism in PANa-induced HepG2 cells. The expression of FANS, p-ACC1, CPT1, ATGL, and AMPKα were evaluated (**a**,**b**,**g**). The levels of FASN (**c**), p-ACC1 (**d**), CPT1 (**e**), ATGL (**f**), and p-AMPKα (**h**) were analyzed in PANa-stimulated HepG2. Con: normal control; PANa: HepG2 was treated with 350 µM PANa for 24 h. HepG2 was incubated with 100 µM KA-8 (KA-8 group), LR-7 (LR-7 group), and PG-7 (PG-7 group) for 4 h, followed by 350 µM PANa treatment for 24 h. *, **, ***, and **** represent *p* < 0.05, <0.01, <0.001, and <0.0001 compared with the PANa group, respectively.

The results presented in Figure 3 demonstrated that the three peptides inhibited fat accumulation and alleviated dyslipidemia induced by PANa treatment, suggesting that their mechanism of action may involve the regulation of lipid metabolism. Fatty acid synthase (FASN), a crucial enzyme involved in fatty acid synthesis, is vital for lipid metabolism [25]. Acetyl-CoA carboxylase (ACC), as one of the primary lipogenic enzymes, plays a significant role in lipid accumulation [26]. To investigate how LR-7, KA-8, and PG-7 influence lipogenesis in HepG2 cells induced by PANa, we measured the levels of FASN and phosphorylated ACC1 (p-ACC1). As depicted in Figure 4, there was a notable increase in FASN expression within the PANa group compared with the Con group while the p-ACC1 levels were significantly reduced (*p* < 0.01). After treatment with LR-7, KA-8, and PG-7, the p-ACC1 levels increased significantly while FASN expression decreased when compared with the PANa group. Among these peptides, LR-7 demonstrated superior efficacy in reducing the FASN levels (59.6%) while enhancing the p-ACC1 concentrations (293.7%).

Carnitine palmitoyl transferase 1 (CPT1) serves as a pivotal enzyme in the process of fatty acid oxidation, whereas adipose triglyceride lipase (ATGL) plays a crucial role in the hydrolysis of triglycerides into fatty acids. Consequently, both CPT1 and ATGL are vital enzymes in lipidolysis and are indispensable for the reduction in blood lipids. The findings presented in Figure 4b indicate that the expression levels of ATGL and CPT1 were markedly diminished in the PANa group relative to the Con group (*p* < 0.05). Conversely, these protein levels exhibited a substantial elevation across all three peptide intervention groups when compared with the PANa group. Notably, among these peptides, LR-7 demonstrated the most significant enhancement in ATGL (148.1%) and CPT1 (134.7%) levels (*p* < 0.0001). AMP-activated protein kinase (AMPK) exerts an inhibitory effect on acetyl-CoA carboxylase (ACC), thereby suppressing the synthesis of fatty acids and triglycerides. Moreover, AMPK mitigates the inhibition of CPT1 activity by decreasing the concentration of malonyl-CoA, thus facilitating fatty acid oxidation. AMPK is recognized as a critical upstream regulator of lipid metabolism [27].

In the present study, PANa was found to attenuate the phosphorylation of AMPKα (p-AMPKα) in HepG2; however, the administration of the three peptides notably ameliorated the reduced levels of p-AMPKα induced by PANa.

### 2.5. Effects of LR-7, KA-8, and PG-7 on Pancreatic Lipase Activity

**Figure 5:**
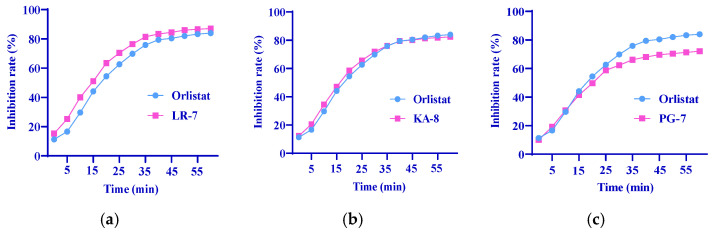
Inhibition of LR-7 (**a**), KA-8 (**b**), and PG-7 (**c**) on pancreatic lipase activity.

Pancreatic lipase is an enzyme produced by the pancreas, and plays a crucial role in fat digestion. By inhibiting pancreatic lipase activity, it is possible to decrease dietary triglyceride digestion and absorption, thereby promoting lipid reduction [28]. This study assessed the pancreatic lipase activity inhibition rates to confirm the lipid-lowering effects of the three peptides. As illustrated in Figure 5, LR-7 exhibited superior inhibitory effects compared with orlistat (the positive control), while KA-8 showed comparable results to orlistat; however, PG-7 was less effective than orlistat. These three peptides showed an inhibition in pancreatic lipase activity, which effectively reduced the digestion and absorption of dietary fat, thereby reducing the level of blood lipids.

## 3. Discussion

Studies have confirmed that oxidative stress and lipid metabolism disorders are significant factors in the occurrence and development of hyperlipidemia Dietary intervention is a crucial auxiliary strategy for the management of hyperlipidemia. Collagen peptides have been extensively used as a nutritional intervention in the treatment of various diseases due to their notable antioxidant, anti-inflammatory, and hypolipidemic activities [17,29]. Our previous study showed that HNCP, an oligopeptide derived from *Harpadon nehereus* bone, attenuated oxidative stress and lowered blood glucose. Another oligopeptide (HNBC), also isolated from *Harpadon nehereus*, was protective against Ang II-stimulated HUVEC injury. Notably, the activation of Nrf2 and AMPK was consistent across all models, suggesting a unified antioxidant pathway, while newly identified lipid-specific targets (e.g., FASN, pancreatic lipase) emphasized the multifunctionality of the peptide. These findings collectively position *Harpadon nehereus* peptides as versatile candidates for managing metabolic syndromes. Therefore, this study aimed to investigate whether three collagen peptides (LR-7, KA-8, and PG-7) extracted from *Harpadon nehereus* bone could ameliorate oxidative stress and lipid metabolism disorders in HepG2 cells, thereby assessing their potential for hyperlipidemia intervention.

Exposure to PANa resulted in a notable decrease in cell viability and increased apoptosis rates; however, treatment with these three peptides led to a substantial recovery in cell numbers. These results indicate that LR-7, KA-8, and PG-7 provide protective benefits against HepG2 cell injury caused by PANa. PANa triggers the buildup of reactive oxygen species (ROS) within cells, resulting in oxidative stress and subsequent cellular injury. Antioxidant enzymes are essential for alleviating oxidative stress [30]. The findings revealed that LR-7, KA-8, and PG-7 significantly decreased the MDA levels while enhancing the activities of SOD, CAT, and GSH-Px. These results suggest that these peptides effectively mitigate oxidative stress in PANa-treated HepG2 cells. Numerous studies have indicated that various peptides can lower oxidative stress by boosting antioxidant enzyme activity in HepG2 cells; those with a higher proportion of hydrophobic amino acids were particularly successful at enhancing this activity [31,32]. Therefore, the higher antioxidant enzyme activities observed in the LR-7 group may be related to the presence of hydrophobic amino acids such as proline (Pro), leucine (Leu), and phenylalanine (Phe). The transcription factor Nrf2 serves as a vital regulator for genes involved in antioxidant responses and electrophilic reactions [33]. Research has shown that hesperetin can reduce hepatic steatosis, alleviate oxidative stress, decrease inflammatory cell infiltration, and mitigate fibrosis through activation of the Nrf2 pathway [34]. In our investigation, we observed a decline in the expression levels of Nrf2, HO-1, and NQO1 following PANa treatment; however, the administration of LR-7, KA-8, and PG-7 led to significant increases in the levels of these proteins. Our findings indicate that LR-7, KA-8, and PG-7 enhance antioxidative capabilities by activating the Nrf2 signaling pathway.

Hyperlipidemia is defined as a metabolic disorder marked by excessive fat accumulation along with elevated levels of TC, TG, and LDL-C [35]. Treatment with PANa significantly enhanced lipid droplet formation alongside a rise in the TG, TCHO, and LDL-C levels. Among the tested peptides, LR-7 notably decreased lipid accumulation while lowering the TG, TCHO, and LDL-C levels in HepG2 cells treated with PANa. These findings validated the lipid-lowering potential of all three peptides but highlighted LR-7 as having the most significant impact. Previous studies have indicated that higher proportions of hydrophobic amino acids within peptides are positively associated with their lipid-lowering capabilities [36]. Therefore, the observed reductions in TG, TCHO, and LDL-C levels within the LR-7 group may also be linked to the presence of Pro, Leu, and Phe.

Lipid metabolism, including lipolysis, lipogenesis, and lipid transport, determines the lipid levels in the body [37]. FASN and ACC are two significant lipid synthetases that play a key role in lipid accumulation. PANa primarily influences hepatic lipogenesis through the modulation of FASN and ACC1 expression [38]. Research has indicated that sesamol (SEM), a kind of natural lignan extracted from sesame oil, can influence lipid accumulation by decreasing the expression levels of FASN and ACC1 [39]. CPT1 and ATGL are closely linked to lipolysis and are crucial for regulating lipid levels. Iso-alpha acids, which are derived from *Humulus lupulus*, a plant cultivated on a large scale across the globe for its use as a raw material in the brewing industry, have been shown to reduce lipid levels by enhancing CPT1 expression in mice with non-alcoholic fatty liver disease (NAFLD) [40]. Recent studies have shown that AMPK regulates both lipolysis and lipogenesis via phosphorylation processes in healthy hepatocytes [41]. In the present study, PANa treatment led to an increase in FASN while simultaneously reducing the ATGL, CPT1, and p-AMPKα levels. Notably, the abnormal expression of proteins associated with lipid metabolism by PANa treatment was significantly reversed by LR-7, KA-8, or PG-7 treatment. Our findings indicate that LR-7, KA-8, and PG-7 modulate lipid metabolism effectively through promoting lipolysis and inhibiting lipogenesis associated with the AMPK pathway, thereby diminishing fat accumulation in HepG2 cells induced by PANa.

Pancreatic lipase, a major enzyme in the breakdown of lipids, is essential for the digestion and absorption of dietary fats [42]. This enzyme gradually converts triglycerides into 2-monoacylglycerol and free fatty acids, which aids in the thorough digestion and absorption of dietary fats [43]. Therefore, the inhibition of pancreatic lipase can reduce the intestinal absorption of triglycerides, thereby preventing hyperlipidemia and obesity [44]. It has been proposed that blocking pancreatic lipase would hinder triglyceride degradation and slow down the entry of fatty acids into systemic circulation and adipose tissue [43]. In this study, we compared the pancreatic lipase inhibitory effects of three peptides with those of orlistat. The results indicated that LR-7 exhibited a stronger inhibitory effect on pancreatic lipase than orlistat, while PG-7 showed less effectiveness compared with orlistat. In summary, these three peptides, particularly LR-7, may lower lipid levels by inhibiting pancreatic lipase activity as well as modulating protein expression related to lipid metabolism. In conclusion, LR-7 exhibits significant antioxidant, lipid metabolism regulation, and lipase activity inhibition, and could be used as a nutritional intervention in the management of hyperlipidemia.

## 4. Materials and Methods

### 4.1. Materials

(PSRILYG, PG-7), (KLHDEEVA, KA-8), and (LALFVPR, LR-7) were isolated and identified in our previous research [20]. HepG2 cells were obtained from the Chinese Academy of Sciences (Shanghai, China). Sodium palmitate (PANa) was sourced from Kunchuang Technology Development Co., Ltd. (Xi’an, China). DMEM was acquired from Gibco Co., Ltd. (Carlsbad, CA, USA). The Oil Red O staining solution along with assay kits for the antioxidant enzymes were purchased from Beyotime Biotechnology (Shanghai, China). Assay kits for blood lipid levels as well as all antibodies used in this study were procured from Nanjing JianCheng Bioengineering Institute (Nanjing, China). All other reagents utilized were of analytical grade.

### 4.2. Cell Cultures

HepG2 cells were cultured in DMEM supplemented with penicillin at a concentration of 100 U/mL, streptomycin at 100 μg/mL, and fetal bovine serum at a volume ratio of 10% under conditions of 37 °C and 5% CO_2_. A total of 2 ×10^4^ cells per well were seeded into a 96-well plate for overnight culture. The upper layer of medium was carefully aspirated, and 200 μL of different concentrations of oligopeptide solution (50 μM, 100 μM, 200 μM) prepared with cell complete medium was added to each well. Twenty-four hours later, the proliferation rate of the cells was measured according to the instructions of the CCK-8 kit, and the appropriate concentration of oligopeptide was screened out.

After incubation, different concentrations (250, 500, 750, 1000 μmol/L) of PANa were added and treated for 24 h. The cell proliferation rates were measured according to the CCK-8 assay instructions.

### 4.3. Cell Viability Assay

A total of 2 × 10^4^ cells/well were inoculated in 96-well plates for culture at 37 °C and 5% CO_2_. Following this period, the cells received treatment with 200 μL medium containing 100 μM LR-7, KA-8, or PG-7 for 4 h before adding 350 μM PANa to each well. After incubation for an additional 24 h, the cell proliferation rates were assessed using the CCK-8 assay protocols.

### 4.4. Observation of Cell Morphology

Cells grown in culture flasks underwent digestion to create a single-cell suspension that was then diluted with fresh medium before being transferred into six-well plates at a final concentration of 4 × 10^5^/well (2 mL/well). Peptides at concentrations of 100 μM were introduced prior to adding PANa for a 4 h incubation. Following another 24 h culture period, cells were examined and photographed using an inverted microscope.

### 4.5. Oil Red O Staining

After incubation, the supernatants were discarded, and 2 mL of Oil Red O stationary solution was added to each well for 20–30 min. The stationary solution was subsequently washed away with 60% isopropyl alcohol. A freshly prepared Oil Red O staining solution was then applied for immersion dyeing lasting 10–20 min. After removing the staining solution with water, a Mayer hematoxylin staining solution was added for counterstaining the nucleus for 1–2 min before being discarded. An Oil Red O buffer solution was applied for 1 min and then discarded. Stained cells were photographed using an Olympus BX41 microscope (Tokyo, Japan) at 100× magnification. The emission wavelength of fluorescence detection was 600 nm.

### 4.6. Determination of Antioxidant Enzymes and Lipid-Related Indexes

After incubation, cells were treated with 100 μM of peptides for 4 h. After removing the media, PANa was added for 24 h of incubation. The levels of catalase (CAT), superoxide dismutase (SOD), glutathione peroxidase (GSH-Px), malondialdehyde (MDA), triacylglycerol (TG), total cholesterol (TCHO), low-density lipoprotein cholesterol (LDL-C), and high-density lipoprotein cholesterol (HDL-C) in the supernatant were determined according to the kit instructions [45].

### 4.7. Western Blot Assay

After treatment, cells were washed with pre-chilled PBS and lysed with ice-cold lysis buffer for 30 min. The supernatant was collected after centrifugation at 12,000× *g* for 10 min and stored at −80 °C. Protein concentration was determined using a BCA protein assay kit. Equal amounts of protein (20–40 µg) were subjected to 10% sodium dodecyl sulfate-polyacrylamide gel electrophoresis (SDS-PAGE) and transferred to PVDF membranes (Polyvinylidene Fluoride, Merck, Darmstadt, Germany). Non-specific binding sites were blocked with 5% bovine serum albumin (BSA) solution at room temperature for 1 h, followed by incubation with the primary antibody at 4 °C overnight. After washing with TBST (3 times, 10 min/time), the membrane was incubated with the secondary antibody at room temperature for 1 h. Following further washing with TBST (3 times, 10 min/time), antibody signals were detected using a Western blot development system. The dilution ratios for the internal reference protein, vascular endothelial injury-related proteins, and oxidative stress-related pathway proteins were prepared according to the manufacturer’s instructions.

### 4.8. Determination of Pancreatic Lipase Activity

A working substrate solution consisting of 4-nitrophenyl palmitate dissolved in sodium acetate solution (containing 1% Triton-X 100, 100 mL, 5 mM, pH 5.0) was prepared. Enzyme powder was dissolved in Tris-HCl buffer (pH 8.0, 37 °C). This mixture was incubated at 4 °C for 2 h before centrifugation at 5000 r/min for 5 min, yielding the supernatant used to prepare a pancreatic lipase solution of 1.2 mg/mL. Orlistat at a concentration of 1 mg/mL in Tris-HCl buffer (pH 8.0; 37 °C) was prepared alongside DMSO to reach a concentration of 350 μg/mL peptide solution. The peptide solution of 100 μM was prepared with Tris-HCl buffer (pH = 8.0, 37 °C). The absorbance was measured at 405 nm, and the inhibition rate was measured in parallel with three holes due to the methodology of Franco et al. [46].

### 4.9. Data Analysis

All experiments were performed three times, and the results were expressed as the mean ± SD. GraphPad Prism 8.0 software was used for one-way ANOVA and comparison between groups.

## 5. Conclusions

This research investigated the effects of three peptides (LR-7, KA-8, and PG-7) derived from bone collagen on PANa-induced hyperlipidemia in HepG2 cells. Our study indicated that all three peptides, particularly LR-7, mitigated the cell damage caused by PANa by enhancing the antioxidant enzyme levels through Nrf2 upregulation. LR-7 demonstrated superior effectiveness in reducing oxidative stress and lipid accumulation compared with KA-8 and PG-7 by modulating the expression of proteins associated with lipid metabolism (FASN, ACC1, ATGL, and CPT1). LR-7 exhibited a stronger inhibitory effect on pancreatic lipase activity than orlistat. Collectively, these results may offer new insights into the potential use of marine peptides from bone collagen as functional foods or therapeutic agents for managing hyperlipidemia. Future studies will aim to further investigate the effects and mechanisms of these three peptides in mice with hyperlipidemia.

## Data Availability

Data are contained within the article.
